# Phytogenic Bioactive Compounds Shape Fish Mucosal Immunity

**DOI:** 10.3389/fimmu.2021.695973

**Published:** 2021-06-18

**Authors:** Joana P. Firmino, Jorge Galindo-Villegas, Felipe E. Reyes-López, Enric Gisbert

**Affiliations:** ^1^ Aquaculture Program, Institut de Recerca i Tecnologia Agroalimentàries (IRTA) Centre de Sant Carles de la Ràpita (IRTA-SCR), Sant Carles de la Ràpita, Spain; ^2^ PhD Program in Aquaculture, Universitat Autònoma de Barcelona, Bellaterra, Spain; ^3^ R&D Technical Department, TECNOVIT – FARMFAES, S.L., Alforja, Spain; ^4^ Faculty of Biosciences and Aquaculture, Nord University, Bodø, Norway; ^5^ Department of Cell Biology, Physiology and Immunology, Universitat Autònoma de Barcelona, Bellaterra, Spain; ^6^ Facultad de Medicina Veterinaria y Agronomía, Universidad de Las Américas, Santiago, Chile; ^7^ Consorcio Tecnológico de Sanidad Acuícola, Ictio Biotechnologies S.A., Santiago, Chile

**Keywords:** immunity, MALT, organosulfurs, terpenes, sustainable aquaculture, teleost, phytogenic additive, TRPV4

## Abstract

Aquaculture growth will unavoidably involve the implementation of innovative and sustainable production strategies, being functional feeds among the most promising ones. A wide spectrum of phytogenics, particularly those containing terpenes and organosulfur compounds, are increasingly studied in aquafeeds, due to their growth promoting, antimicrobial, immunostimulant, antioxidant, anti-inflammatory and sedative properties. This trend relies on the importance of the mucosal barrier in the fish defense. Establishing the phytogenics’ mode of action in mucosal tissues is of importance for further use and safe administration. Although the impact of phytogenics upon fish mucosal immunity has been extensively approached, most of the studies fail in addressing the mechanisms underlying their pharmacological effects. Unstandardized testing as an extended practice also questions the reproducibility and safety of such studies, limiting the use of phytogenics at commercial scale. The information presented herein provides insight on the fish mucosal immune responses to phytogenics, suggesting their mode of action, and ultimately encouraging the practice of reliable and reproducible research for novel feed additives for aquafeeds. For proper screening, characterization and optimization of their mode of action, we encourage the evaluation of purified compounds using *in vitro* systems before moving forward to *in vivo* trials. The formulation of additives with combinations of compounds previously characterized is recommended to avoid bacterial resistance. To improve the delivery of phytogenics and overcome limitations associated to compounds volatility and susceptibility to degradation, the use of encapsulation is advisable. Besides, newer approaches and dedicated methodologies are needed to elucidate the phytogenics pharmacokinetics and mode of action in depth.

## Introduction

Sustainable food supply to feed the demand of the projected world population by 2050 is a challenge, in which aquaculture is predicted to be the main source of aquatic dietary proteins. Such growth will unavoidably involve the implementation of innovative aquaculture production strategies, targeting issues related to effective health management and animal welfare (1). In this regard, the development and application of functional feeds represent a sound strategy to improve aquaculture systems, since they provide functional health benefits to animals beyond their nutritional value (2). In this scenario, phytogenics, also known as phytobiotics, are defined as environmentally friendly plant-derived bioactive compounds used as functional feed additives that show positive effects on animal growth and health. Phytogenic often comprise aromatic plants extracts, and essential oils characterized by its richness in biologically active compounds (3, 4). In farmed fish, a wide spectrum of phytogenics have been increasingly studied mainly due to their wide repertoire of properties, including growth promotion, and antimicrobial, immunostimulant, antioxidant, anti-inflammatory and sedative activities (5). In particular, phytogenics derived from Lamiaceae family and *Allium* sp. are among the most commonly studied and administrated plant-based additives (6, 7). Nonetheless, the complexity of the mechanisms of action and the pharmacological effects promoted by the diverse bioactive compounds present in such plants, along with their frequently observed synergistic behavior (8), often limits the full understanding of their biological activity (9).

Since outbreaks of fish diseases are one of the main constrains for the progress of the aquaculture sector (10), the inclusion of phytogenics’ in aquafeeds is achieving significant attention at a global scale. The impact of phytogenics upon fish systemic immunity has been extensively tested in the past (5, 9). However, an increasing trend to evaluate phytogenics’ impact upon the mucosal immunity has been gained importance in recent years, which is mainly attributed to the importance of the mucosal barrier in the fish defense against variated threats and, potential colonization and invasion by pathogenic organisms (11). In contrast, most of the studies evaluating the effect of phytogenics in fish systemic immunity are only supported by a selection of repetitive primary analyses (*i.e.*, lysozyme, bacteriolytic and complement activities, immunoglobulins, oxidative stress enzymes, etc.) serving only as proxies, that only provide a snapshot of the effects of the evaluated feed additive on the organism. These approaches do not allow elucidating their mode of action at cellular and molecular levels. This is of special relevance when dealing with functional feed additives with potential pharmacological properties, as their standardized use mainly depends on the proper understanding of their regulatory properties in the immune system.

Phytogenic administration has the potential to regulate the mucosal barrier function by means of several molecular mechanisms, in which the phytogenic bioactive compounds interact with cellular transcription factors and metabolic cascades. Therefore, the modulation of the expression of genes coding for immune relevant molecules alter the mucosal protective characteristics and their immunological status (12–16). Besides, the immune system influences the regulation and composition of the microbiota and *vice versa*, an interaction that plays a determinant role in the maintenance of the mucosal integrity and functionality (17, 18). Hence, both the improvement of the mucosal barrier characteristics and the modulation of the microbiota are central targets for the development of new phytogenic additives, while understanding their mode of action at cellular and molecular levels is critical for elucidating their benefits to the host.

Given the extended literature available on plant-based functional additives and the significance of the mucosal immunity described above, our review efforts focus exclusively on the physiological and immunological responses achieved by the most studied fish mucosal tissues, intestine, gills and skin, of organisms fed with phytogenics of the Lamiaceae family and *Allium* sp. In the first part, we present a thorough description on their main bioactive compounds and relevant biological properties. Then, the immunomodulatory properties and the mechanisms they can trigger on the fish mucosal tissues are explored and further potential mechanisms hypothesized. Finally, research gaps and constrains for the development of applicable phytogenic-based additives are discussed. Overall, the information presented herein aims to provide clear insights on the fish mucosal immune response dietary treated with phytogenics, propose viable mechanisms for exploiting them, and ultimately encourage the practice of reliable and reproducible research for the development of novel feed additives to be used as sustainable and safe prophylactic strategies for aquaculture.

## Fish Mucosal Immunity at a Glance

The mucosal barrier constitutes the fish first line of defense against the surrounding environment and potential pathogens. Fish mucosal tissues are particularly characterized by a mucosa-associated lymphoid tissue (MALT), harboring diverse myeloid and lymphoid cells that are responsible for the host protection against pathogens and antigens, while tolerating beneficial symbiont colonization to maintain mucosa homeostasis (19–21). Six different MALTs have been described so far in teleosts. The gut-associated lymphoid tissue (GALT), the gill-associated lymphoid tissue (GIALT), the skin-associated lymphoid tissue (SALT), the nasopharynx-associated lymphoid tissue (NALT) and, the more recently characterized the buccal, and pharyngeal MALTs (22). Other mucosal immune systems have been hypothesized and are currently under study (23). Despite the existence of others, the GALT, GIALT and SALT are the most studied and well characterized MALTs and therefore selected as target in this review.

Among the extensive cell types with immune capacity coexisting in the fish body, upon sensing the presence of pathogenic or commensal microbe-associated molecular pattern (MAMP) a downstream signaling response mediated through pattern recognition receptors (PRRs) immediately takes place. So far, several piscine PRRs have been identified, being the toll-like receptors (TLR), NOD-like receptors (NLR) and retinoic acid-inducible gene I (RIG-I)-like receptors (RLRs) the best characterized (24, 25). Epithelial and endothelial cells together with the professional phagocytes, represented by macrophages, granulocytes and dendritic cells, are the first responders against MAMPs formerly sensed by PRRs. Phagocytosis contributes to both pathogen clearance and subsequent antigen presentation to other immune cells by the membrane Major Histocompatibility Complex (MHC) class II peptide complex (26). In most, but not all teleost fish, the peptide-MHC II complex activates naive CD4^+^ T cells expressing antigen-specific T cell receptors (TCR) in their surface. Recognition of this complex stimulates the dedicated CD4^+^ cells activation and differentiation into T helper cell subsets possessing inflammatory cytokines secreting capacity that further coordinate the adaptive response together with B cells (27, 28). Interestingly, while CD4^+^ T helper lymphocytes are mainly present in the gut *lamina propria*, the cytotoxic CD8^+^ cells are the dominant intraepithelial resident immunocytes (29–31).

In fact, both T and B lymphocytes are abundantly present in fish mucosal tissues (32). Interestingly, the phagocytic and bacterial-killing abilities of B cells in fish have been fairly introduced in the past (33). However, their antigen presentation mechanisms mediated by MHC II and costimulatory molecules (CD80/CD86 and CD83) to prime naïve CD4^+^ T-cells, produce IgM, IgT, and eventually IgD plasmablasts -a major lymphocyte population in the gut, gill and at some extent the skin-, have just been recently addressed (34, 35). The IgT, the teleost specialized mucosal immunoglobulin analogous to mammalian IgA (36), plays a critical role in the clearance of mucosal pathogens and the preservation of microbiota homeostasis through immune exclusion (11). Although extremely important in mucosal defense, not all teleosts present IgT/IgD, which suggests the existence of alternative mucosal immune systems (23).

For instance, the complete IgM and IgT sequences in their membrane and soluble forms have been reported and characterized for the first time in a perciform, the gilthead sea bream (*Sparus aurata*) (37). Interestingly, this study demonstrated that virus and bacteria trigger the mucosal immune response by promoting the activation of IgT in seabream. Although, diets with fish oil replacement by ones from plant origin inhibited the IgT up-regulation upon intestinal parasitic challenge, which was related to a worse disease outcome. These results evidenced that mucosal immunoglobulins can be significantly affected by dietary treatments, which highlights the necessity of testing this response case by case.

Although characterized by common cellular components, immune mediators and immune mechanisms, the different composition, organization and functions of MALTs may vary according to each tissue intrinsic and external environmental factors (38), changes that may be associated to the fish species considered. Besides, the microbiota also stands as a relevant component of the mucosal barrier, displaying an antagonistic behavior against invading “hostile” microorganisms and directly participating in the immune responses through the complex host-microbiota crosstalk at the mucosal interface (17, 18). Therefore, the selective manipulation of the microbiota by means of nutritional approaches has been previously proposed as a viable alternative to modulate mucosal responses (39).

The mucosal tissues are intrinsically characterized by mucus secreting cells, such as goblet cells (40). Beside playing important roles in intra- and interspecific ecological interactions (41) and being a key component that ensures host-microbiota homeostasis (42, 43), the secreted semipermeable mucus represents the first challenge that pathogens have to overcome in order to interact with the host. Its complex composition encompasses a matrix of glycoproteins, the mucins that confer the mucus its structure, and a wide variety of humoral immune factors, such as lysozymes, complement, lectins, proteolytic enzymes, antimicrobial peptides, immunoglobulins, among others (41). Moreover, the mucus is continuously secreted and replaced (44); this continuous secretion aims to prevent pathogen attachment and interaction with the host. Therefore, the presence of a mucus layer is fundamentally involved in the regulation of the mucosal immune system, not only as a protective physical and chemical barrier, but also acting as a vehicle for mucins and humoral immune factors from the inside out. Both goblet cells (13, 15) and mucus composition (45) are highly susceptible to be manipulated through dietary strategies, which opens a wide range of possibilities when to design and apply new functional feed additives.

## Lamiaceae Family and *Allium* sp. Among the Most Studied Aromatic Plants Used as Phytogenics in Aquaculture

In nature, plant secondary metabolites have functional roles independent from plant growth and development; thus, protecting plants from herbivore and pests, or acting as chemoattractants for pollinators (46). These bioactive compounds broadly found in aromatic plants are usually present as mixtures, mainly represented by phenolics and terpenes that are chemically characterized by their aromatic rings (3). Therefore, their benefits as dietary supplements are subject to the variability and complexity of the aromatic compounds mixture, apart from their synergistic effect, their origin, the dietary inclusion level and their pharmokinetics (47).

In particular, phytogenics derived from Lamiaceae family and *Allium* sp. are among the most widespread administrated plant-based additives in aquaculture (48) and livestock (6, 7). These compounds are used for their recognized growth promoting, antimicrobial, immunostimulant, antioxidant, anti-inflammatory and sedative properties. Although they can be found worldwide, some representatives of this group of aromatic plants (*i.e.*, oregano, thyme, basil, menthe, rosemary, sage, marjoram, garlic and onion, among others) are particularly present and traditionally consumed in the Mediterranean area and appreciated in terms of human nutrition and therapy (49, 50). The health-promoting properties of these aromatic plants have been extensively reviewed in different aquaculture species (5, 48, 51–55). However, most of the studies dealing with these functional feed additives were only focused in physiological or biochemical responses, but few of them have elucidated the cellular and molecular mechanisms underlying their immunostimulatory capacity.

While the existent information about the inherent effect of these phytogenics upon immune cells is limited under *in vitro* conditions, numerous *in vivo* studies have demonstrated an improvement of the fish mucosal immune responses following their administration. A refined complementary search through Web of Science, PubMed and Google Scholar was performed in this review. Until March 2021, 62 publications reporting the nutritional effects of Lamiaceae family and *Allium* sp., or related bioactive compounds, upon fish mucosal responses were retrieved and their results summarized in **Table 1**. Importantly, most of them were published in the last year; thus, evidencing the current increasing interest for research on phytogenics targeting mucosal tissues. From the overall bibliographic search results, few publications felt within the objective of the present review and described the cellular or molecular mechanisms underlying fish mucosal immune responses to phytogenics’ administration. Studies reporting the application of plant extracts or related compounds as bath treatments or evaluating bactericidal or antiparasitic effects *in vitro* were excluded from the selection as this review is just focused on the mucosal immune mechanisms. Furthermore, **Table 1** omits those results out of the mucosal immunity context, including systemic immunity-related results, non-immune digestive parameters or other complementary analysis performed within each study. Although such variables are extensively used as key performance indicators in such studies, their relevance in terms of supporting and/or establishing the mode of action of phytogenics is questionable and out of the scope for this review. Finally, blends with other components besides the selected group of plants –or associated bioactive compounds such as terpenes or organosulfurs– were excluded as well.

**Table 1:** Extended summary of the current available literature on nutritional effects of Lamiaceae family and *Allium* sp. derived phytogenics upon fish mucosal immune response.

Phytogenic plant origin	Supplemented form	Inclusion dosage(s) tested	Period of administration	Main bioactive components (≤ 3)	Fish species	Performance	Mucosal parameters evaluation	Key benefits summary	Reference
**Lamiaceae family**									
Oregano (*Origanum vulgare*)	Powder	0.5%, 1.0% and 2.0%	8 weeks	N/I	Zebrafish (*Danio rerio*)	↑ Final weight ↑ Weight gain ↓ FCR ↑ SGR ↑ Survival against *A. hydrophila*	↑ Skin mucus lysozyme activity ↑ Skin mucus alkaline phosphatase activity ↑ Skin mucus total Ig ↑ Skin mucus protease activity ↑ Skin mucus total protein	Beneficially affects the skin mucus immune parameters, growth performance and survival against pathogenic bacterial challenge	Rashidian, Boldaji (56)
Oregano (*Origanum vulgare*)	Powder	0.5% and 1.0%	15 and 30 days	N/I	Gilthead seabream (*Sparus aurata*)	No effect	↑ Skin mucus IgM ↑ Skin mucus bactericidal activity against *P. damselae*	Oregano improves humoral immunity and increases the antibacterial activity of skin mucus	Beltrán, Gonzalez Silvera (57)
Oregano (*Origanum vulgare*)	Ethanolic extract	0.2% and 0.5%	60 days + 7 days *A. hydrophila* challenge	N/I	Nile tilapia (*Oreochromis niloticus*)	↑ Final weight ↑ Weight gain ↓ FCR ↑ SGR ↑ Survival rate ↑ Survival against *A. hydrophila*	↑ Skin mucus total Ig	Can effectively improve the fish growth, health, and immune status	Mohammadi, Rafiee (58)
Oregano (*Origanum vulgare*)	Powder	5.0, 10.0, 15.0 and 20.0 g kg^-1^	8 weeks	Carvacrol and thymol (Commercial product)	Common carp (*Cyprinus carpio*)	↑ Final weight ↑ Weight gain ↑ SGR	↑ Intestine villus height ↑ Intestine villus width ↑ Intestine crypt depth	Dose-dependent enhancement of intestinal morphometry, which subsequently lead to improvement of nutrients absorption	Abdel-Latif, Abdel-Tawwab (59)
Oregano (*Origanum vulgare*)	Essential oils	0.75, 1.5, 2.25 and 3.0 g kg^-1^	64 days	Carvacrol, thymol and p-cymene	Nile tilapia (*Oreochromis niloticus*)	No effect	↑ Intestine villus height	Increases intestinal villus size	Heluy, Ramos (60)
Oregano (*Origanum vulgare*)	Essential oils	0.5, 1.5 and 4.5 g kg^-1^	8 weeks + 7 days *A. hydrophila* challenge	N/I	Koi carp (*Cyprinus carpio*)	↑ Survival against *A. hydrophila*	↓ TNF-a and TGF-b gene expression in intestine ↑ Actinobacteria phylum, and *Propionibacterium*, *Brevinema* and *Corynebacterium* genera ↓ Bacteroidetes phylum and *Vibrio* genus No effect on bacterial alpha diversity	Presents immunomodulatory effects and enhances disease resistance. Also beneficially alters the gut bacterial community composition of fish	Zhang, Wang (61)
Oregano (*Origanum vulgare*)	Powder	0.5% and 1.0%	30 days + cypermethrin exposure	Carvacrol and thymol (Commercial product)	Common carp (*Cyprinus carpio*)	N/I	↓ Gill histopathologic lesions ↓ Gill proliferating cell nuclear antigen (PCNA) and caspase-3 immune positive cells	Protective roles against the adverse effects of cypermethrin, enhancing recovery from the exposure	Khafaga, Naiel (62)
Oregano (*Origanum vulgare*)	Essential oils	0.01%, 0.02%, 0.05% and 0.10%	24 days + 28 days *I. salmonis* and *T. truttae* challenge (Total 52 days)	Carvacrol, p‐cymene and γ‐terpinene	Chum salmon (*Oncorhynchus keta*)	↑ feed efficiency ↓ *I. salmonis* infection ↓ *T. truttae* infection ↓ cumulative mortality	Carvacrol content detected in the skin of fish fed the oregano supplemented diet	Preventive effects against *I. salmonis* and *T. truttae* and suggests the possibility that its anti‐parasitic action is attributable to the bioactive component emergence through the skin	Mizuno, Urawa (63)
Oregano (*Origanum vulgare*)	Essential oils	0.5, 1.0, 1.5, 2.0 and 2.5 g kg^-1^	90 days	N/I (Commercial product)	Yellow tail tetra (*Astyanax altiparanae*)	N/I	↑ Intestine villus length ↑ Intestine villus width ↑ Intestine absorption area ↑↓ Intestine goblet cells number	Promotes increased absorption surface area and modulates the number of goblet cells involved in protecting the intestinal mucosa	Ferreira, Caldas (64)
Marjoram (*Origanum majorana*)	Ethanolic extract	0.1, 0.2 and 0.4 g kg^-1^	60 days + 10 days *A. hydrophila* challenge	N/I	Common carp (*Cyprinus carpio*)	↑ Final weight ↑ Weight gain ↓ FCR ↑ SGR ↑ Survival against *A. hydrophila*	↑ Skin mucus alkaline phosphatase ↑ Skin mucus total Ig ↑ Skin mucus lysozyme activity ↑ Skin mucus alternative complement (ACH50) activity	Increase fish skin mucosal immunity and performance	Yousefi, Ghafarifarsani (65)
Thyme (*Thymus vulgaris*)	Essential oils	500 ppm	30 days + thiamethoxam exposure	Thymol, p-cymene and γ-terpinene	African catfish (*Clarias garipenus*)	N/I	↓ Gill histopathologic lesions	Mitigate the thiamethoxam induced toxicity	El Euony, Elblehi (66)
Thyme (*Thymus vulgaris*)	Aqueous extract	5.0, 10.0 and 20.0 g kg^-1^	2 weeks + oxytetracycline	N/I	Rainbow trout (*Oncorhynchus mykiss*)	No effect	↑ Intestine antioxidant enzymes (SOD, CAT, GPx and GST) activity ↓ Intestine levels of the oxidative stress marker malondialdehyde	Mitigate adverse effects of oxytetracycline and improve the fish immune responses	Hoseini and Yousefi (67)
Thyme (*Thymus vulgaris*)	Essential oils	0.1%, 0.5%, and 1%	15 days	Thymol, o-cymene and carvacrol	Nile tilapia (*Oreochromis niloticus*)	N/I	No effect upon the population of beneficial Bacillus bacteria in the gut	Stimulated the cellular components of the non-specific immune response without deleterious effects on the general health of the fish or the intestinal tract	Valladão, Gallani (68)
Thyme (*Thymus vulgaris*)	Essential oils	0.005, 0.010 and 0.02 g kg^-1^	5 weeks	Thymol, p‐cymene and linalool	Rainbow trout (*Oncorhynchus mykiss*)	No effect	No effect upon the allochthonous microbiota profile	No toxic effects do not significantly alter the intestinal contents bacterial populations	Navarrete, Toledo (69)
Spanish thyme (*Thymus zygis* subsp. *gracilis*)	Essential oils	0.001, 0.002, 0.003 and 0.004 g kg^-1^	12 weeks	Thymol, p‐cymene and carvacrol	Gilthead seabream (*Sparus aurata*)	No effect	↑ Anterior intestine lymphocyte aggregates in the lamina propria at low dose ↓ Anterior intestine lymphocyte aggregates in the lamina propria at high doses	Dose-dependent immuno‐modulatory effect upon the intestine	Hernandez, Garcia (70)
Lemon balm (*Melissa officinalis*)	Ethanolic extract	0.2% and 0.5%	60 days + 7 days *A. hydrophila* challenge	N/I	Nile tilapia (*Oreochromis niloticus*)	↑ Final weight ↑ Weight gain ↓ FCR ↑ SGR ↑ Survival rate ↑ Survival against *A. hydrophila*	↑ Skin mucus total Ig ↑ Skin mucus lysozyme activity ↑ Skin mucus protease activity ↑ Skin mucus alternative complement (ACH50) activity	Can effectively improve the fish growth, health, and immune status	Mohammadi, Rafiee (58)
Peppermint (*Mentha piperita*)	Powder	2.0, 3.0, and 4.0 g kg^-1^	8 weeks	N/I	Caspian roach (*Rutilus caspicus*)	↑ Final weight ↑ Weight gain ↓ FCR ↑ SGR ↓ Daily intake rate	↑ Secretion of skin mucosal protein pattern bands; higher lysozyme band intensity in particular ↑ Skin mucus lysozyme activity ↑ Skin mucus alkaline phosphatase activity ↑ Skin mucus soluble protein	Act as a growth promoter and immunostimulant	Paknejad, Hosseini Shekarabi (71)
Peppermint (*Mentha piperita*)	Essential oils	0.1 and 0.25 g kg^-1^	7, 14, 30 and 60 days	Menthol, mentone and 1,8-cineole	Nile tilapia (*Oreochromis niloticus*)	N/I	↑ Intestine intraepithelial lymphocytes	Show benefits in terms of intestinal health and on immune parameters	Valladão, Gallani (72)
Peppermint (*Mentha piperita*)	Ethanolic extract	1.0%, 2.0% and 3.0%	8 weeks	N/I	Rainbow trout (*Onchorhynchus mykiss*)	↑ Survival against *A. hydrophila*	↑ Skin mucus antibacterial activity against *S. iniae*, *Y. ruckeri*, *A. hydrophila* and *L. garviea*	Triggers the immune system of rainbow trout against *Y. ruckeri*	Adel, Pourgholam (73)
Peppermint (*Mentha piperita*)	Ethanolic extract	1.0%, 2.0% and 3.0%	56 days	N/I	Caspian kutum roach (*Rutilus frisii kutum*)	↑ Weight gain ↓ FCR ↑ SGR	↑ Skin mucus antibacterial activity against *S. iniae*, *Y. ruckeri*, *L. monocytogenes* and *E. coli* ↑ Skin mucus protein level ↑ Skin mucus alkaline phosphatase activity	Increases the mucosal immune parameters and performance of fry in a dose dependent manner	Adel, Amiri (74)
Peppermint (*Mentha piperita*)	Ethanolic extract	1.0%, 2.0% and 3.0%	8 weeks	N/I	Caspian brown trout (*Salmo trutta caspius*)	↑ Weight gain ↓ FCR ↑ SGR	↑ Skin mucus protein level ↑ Skin mucus lysozyme activity ↑ Skin mucus alkaline phosphatase activity	Promote growth performance and have immunostimulant properties	Adel, Safari (75)
Horsemint (*Mentha longifolia*)	Ethanolic extract	2.0%, 4.0% and 6.0%	8 weeks + *Y. ruckeri* challenge	N/I	Caspian kutum roach (*Rutilus frisii kutum*)	↑ Weight gain ↓ FCR ↑ SGR ↑ Survival rate	↑ Skin mucus protein level ↑ Skin mucus lysozyme activity ↑ Skin mucus alkaline phosphatase activity ↑ Skin mucus protease activity ↑ Skin mucus esterase activity ↑ Skin mucus antibacterial activity against *S. iniae, Y. ruckeri, A. hydrophila* and *L. garvieae*	Improve growth performance and boost fish immune response in a dose‐related manner	Gholamhosseini, Adel (76)
Horsemint (*Mentha longifolia*)	Hydroalcoholic extract	0.1%, 0.2% and 0.3%	4 weeks + 10 days *Y. ruckeri* challenge	N/I	Rainbow trout (*Onchorhynchus mykiss*)	↑ Survival against *Y. ruckeri*	↑ Secretion of skin mucosal protein pattern bands; higher lysozyme band intensity in particular	Dose‐related positive effect on immunogenicity and increased resistance to bacterial disease	Heydari, Firouzbakhsh (77)
Thumbai (*Leucas aspera*)	Powder	1.0, 2.0, 4.0 and 8.0 g kg^-1^	45 days + 15 days *S. agalactiae* challenge	N/I	Nile tilapia (*Oreochromis niloticus*)	↑ Weight gain ↓ FCR ↑ SGR ↑ Survival against *S. agalactiae*	↑ Skin mucus lysozyme activity ↑ Skin mucus peroxidase activity	Increase skin mucosal immune parameters, performance and survival against bacterial infection	Kurian, Van Doan (78)
Shirazi thyme (*Zataria multiflora*)	Hydroalcoholic extract	2.0 g kg^-1^	56 days	Thymol and carvacrol? (N/I)	Rainbow trout (*Oncorhynchus mykiss*)	↑ Survival rate	↑ Skin mucus bactericidal activity against *A. hydrophila* ↑ Skin mucus lysozyme activity	Increase skin mucosal immunity	Mirghaed, Hoseini (79)
Shirazi thyme (*Zataria multiflora*) + Rosemary (*Rosmarinus officinalis*)	Powder (1:1) + aflatoxin B1	40 g kg^-1^ (20 g kg-1 each)	12 weeks	N/I	Common carp (*Cyprinus carpio*)	No effect	No effect	Do not prevent intestinal tissue lesions induced by aflatoxin B1	Tasa, Imani (80)
Rosemary (*Rosmarinus officinalis*)	Aqueous extract	10, 20, 40, 80 and 100 ml/100 g	20 days	1,8-Cineole	Common carp (*Cyprinus carpio*)	N/I	↑ Skin mucus level of 1,8-Cineole dose-dependent No effect upon intestine histopathology	High volume of extracts might promote hepatic toxicity	Zoral, Ishikawa (81)
Rosemary (*Rosmarinus officinalis*)	Powder	0.6, 1.2, 1.8 and 2.4 g kg^-1^	4 and 12 weeks	Carnosic acid and carnosol (1:1)	Gilthead seabream (*Sparus aurata*)	No effect	No effect	The histological examination of the intestine showed no aspects that might pose problems for absorption, or any immune system disorder associated with the intestine	Hernandez, Garcia Garcia (82)
Oliveria (*Oliveria decumbens*)	Essential oils and/or hydroethanolic extract	0.01%, 0.1% and 1.0%	60 days + 14 days *S. iniae* challenge	γ-terpinene, carvacrol and thymol	Nile tilapia (*Oreochromis niloticus*)	↑↓ Survival against *S. iniae*	No effect	Increase fish survival 14 days after challenge with *S. iniae*	Vazirzadeh, Jalali (83)
Clove basil (*Ocimum gratissimum*)	Ethanolic extract	5.0, 10.0, and 15.0 g kg^-1^	12 weeks + 14 days *L. monocytogenes* challenge	N/I	African catfish (*Clarias gariepinus*)	↑ final weight ↑ weight gain ↑ SGR ↑ Feed intake ↑ survival against *L. monocytogenes*	↑ intestine villus length ↑ intestine villus width ↑ intestine absorption area	Improve the fish performance, health, and immune response	Abdel-Tawwab, Adeshina (84)
Clove basil (*Ocimum gratissimum*)	Essential oils	0.5%, 1.0% and 1.5%	55 days + 10 days *S. agalactiae* challenge	1,8-cineole, eugenol and β-selinene	Nile tilapia (*Oreochromis niloticus*)	N/I	↑ Intestine villus height ↑ Intestine goblet cells number ↓ Gill epithelial detachment in the secondary lamellae ↓ Gill congestion at the base of the secondary lamellae	Ameliorate tissue damages, even in situations of infection	Brum, Cardoso (85)
American basil (*Ocimum americanum*)	Essential oils	0.25, 0.5, 1.0 and 2.0 g kg^-1^	7 weeks	Linalool, eugenol and 1,8-cineole	Red drum (*Sciaenops ocellatus*)	No effect	↑ Stomach lysozyme activity No effect upon the intestinal microbial community	Different supplementation levels do not influence growth performance and intestinal microbial community; however, show effects on immunological responses	Sutili, Velasquez (86)
Savory (*Satureja khuzestanica*)	Powder	1%	45 days	N/I	Common carp (*Cyprinus carpio*)	N/I	↑ Intestinal lactic acid bacteria	Improves intestinal health	Mousavi, Mohammadiazarm (87)
***Allium* sp.**									
Garlic (*Allium sativum*)	Aqueous extract	0.10, 0.15, and 0.20 ml kg^-1^	80 days	N/I	Guppy (*Poecilia reticulata*)	No effect	↑ Skin mucus lysozyme ↑ Skin mucus alternative complement ↑ Skin mucus total Ig ↑ Skin mucus alkaline phosphatase	Administration of 0.15 mL of garlic extract per kg feed is suggested to obtain optimal skin mucus immunity	Motlag, Safari (88)
Garlic (*Allium sativum*)	Oil	50 µl kg^-1^	28 days + exposure to silver nanoparticles	N/I	Rohu (*Labeo rohita*)	N/I	↓ Gill oxidative stress enzymes activity ↓ Gill histopathologic lesions	Amelioration of silver nanoparticles-induced oxidative stress and histoprotective effects	Khan, Qureshi (89)
Garlic (*Allium sativum*)	Powder	0.5 g and 1.0 g/100 g	2 months + 2 weeks *S. iniae* challenge	N/I	Nile tilapia (*Oreochromis niloticus*)	↑ Survival against *S. iniae*	↑ Anterior intestine transcriptional levels of interleukin genes (IL‐10 and IL‐17F) ↑ OTU counts for the phylum of *Proteobacteria* and *Tenericutes*	Could be effective in the prevention of *S. iniae* infection in fish	Foysal, Alam (90)
Garlic (*Allium sativum*)	Powder	5.0%, 10.0% and 20.0%	14 or 28 or 32 days + *C. irritans* challenge	Allicin (1.25 mg/g)	Guppy (*Poecilia reticulata*)	↓↑ Gills and caudal fin *C. irritans* infection	N/I	No clear preventative effect against *C. irritans*	Kim, Fridman (91)
Garlic (*Allium sativum*)	Powder	1.0%, 1.5% and 2.0%	120 days		Rainbow trout (*Oncorhynchus mykiss*)	↑ Weight gain ↑ SGR	↓ Bacterial diversity and richness ↑*Deefgea*, *Mycoplasma*, *Exiguobacterium* and *Clostridium* genera ↓↑ *Aeromonas* genus	Beneficial in terms of promoting growth and inducing changes in the intestinal microbiota in a dose-dependent manner	Büyükdeveci, Balcázar (92)
Garlic (*Allium sativum*)	Powder	5.0, 10.0 and 15.0 g kg^-1^ diet	8 weeks	N/I	Caspian roach (*Rutilus rutilus*)	↑ Weight ↑ Growth rate	↑ Skin mucus antibacterial activity against *S. faecium*, *M. luteus*, *S. marcescens* and *E. coli* ↑ Skin mucus protein level ↑ Skin mucus alkaline phosphatase activity	Beneficially affects the skin mucus immune parameters and growth performance	Ghehdarijani, Hajimoradloo (93)
Garlic (*Allium sativum*)	Lyophilized	2.0%	21 days + cadmium exposure	N/I	Prussian carp (*Carassius gibelio*)	N/I	↓ Gill histopathologic lesions	Shows chelating and antioxidant potential	Nicula, Dumitrescu (94)
Onion (*Allium cepa*)	Powder	1%	45 days	N/I	Common carp (*Cyprinus carpio*)	N/I	↑ Intestinal lactic acid bacteria	Improves intestinal health	Mousavi, Mohammadiazarm (87)
Onion (*Allium cepa*)	Ethanolic extract	0.5%, 1.0%, 1.5% and 2.0%	12 weeks	N/I	African sharptooth catfish (*Clarias gariepinus*)	No effect	↓ Intestine villus length ↑ Intestine villus width ↑ Intestine absorption area ↑ Intestine cryptal depth	Increase the digestive and absorptive capacity of the intestine	Bello, Emikpe (95)
Mongolian Wild Onion (*Allium mongolicum*)	Ethanolic extract	0.04 g kg^-1^	4 weeks + chromium (Cr) exposure	Flavonoids ≥90% (HPLC)	Grass carp (*Ctenopharyngodon idella*)	N/I	↓ Intestine and gill Cr accumulation ↓ Gill malondialdehyde content ↓ Gill protein carbonyl content ↑ Intestine lysozyme activity ↑ Intestine complement 3 levels ↑ Intestine and gill tight junction proteins gene expression ↑↓ Intestine and gill NF-κB signaling pathway gene expression	Decrease in Cr-accumulation, oxidative stress, immunosuppression and inflammatory response following Cr exposure	Zhao, Yuan (96)
**Single bioactive compounds**									
Thyme (*Thymus vulgaris*)*	Ethanolic solution	0.15, 0.3, 0.45, 0.6, 0.75 g kg^-1^	56 days + 14 days *A. veronii* challenge	Thymol (commercial product)	Snakehead fish (*Channa argus*)	↑ Final weight ↑ Weight gain ↑ SGR ↑ Protein efficiency ratio ↓ FCR ↑ Survival *to A. veronii*	↑ Intestine SOD, CAT, GSH-Px activities ↓ Intestine malondialdehyde content ↑ IL-10 and TGF-b gene expression in intestine ↓ HSP70, TNF-a, IL-1b and IL-8 gene expression in intestine	Adequate dietary supplementation can effectively enhance the growth, antioxidant status, immune response and disease resistance	Kong, Li (97)
Thyme (*Thymus vulgaris*)*	N/I	0.1, 0.2 and 0.3 g kg^-1^	60 days + 8 days *A. hydrophila* challenge	Thymol (commercial product)	Grass carp (*Ctenopharyngodon idella*)	↑ Final weight ↑ Survival against *A. hydrophila*	↑ Gill enzymes of the phosphotransfer network: cytosolic and mitochondrial creatine kinases, adenylate kinase activities and ATP levels in infected fish ↓ Gill ROS levels in infected fish	Favors weight gain and fish longevity. Prevents *A. hydrophila* induced branchial bioenergetics. High concentrations deserve attention because of side-effects	Morselli, Baldissera (98)
Thyme (*Thymus vulgaris*)*	Crystals	0.5 g kg^-1^	70 days	Thymol (99% purity; commercial product)	Nile tilapia (*Oreochromis niloticus*)	↑ SGR ↑ Protein efficiency ratio	↓ Intestine total aerobic and anaerobic counts	Improve some performance parameters and negatively modulates intestinal microbial communities. Demonstrates a notable synergistic interaction with chitosan nanoparticle with beneficial effects	El-Naby, Al-Sagheer (99)
Thyme (*Thymus vulgaris*)*	N/I	1.0 g kg^-1^	56 days	Thymol (commercial product)	Rainbow trout (*Oncorhynchus mykiss*)	↓ FCR	↓ Intestine culturable anaerobe bacteria	Modulated intestinal microbial communities disfavoring total anaerobes	Giannenas, Triantafillou (100)
Oregano (*Origanum vulgare*)*	N/I	1.0 g kg^-1^	56 days	Carvacrol (commercial product)	Rainbow trout (*Oncorhynchus mykiss*)	↓ FCR	↓ Intestine culturable anaerobe bacteria	Modulated intestinal microbial communities disfavoring total anaerobes	Giannenas, Triantafillou (100)
Chinese skullcap (*Scutellaria baicalensis*)*	Powder	0.4, 0.8 and 1.6 g kg^-1^	60 days + H_2_O_2_ challenge	Baicalin (80% purity, commercial product)	Nile tilapia (*Oreochromis niloticus*)	↓ FCR	↑ Gill glutathione level ↑ Gill total antioxidant capacity	Improves feed efficiency, enhance antioxidative ability and alleviate oxidative stress	Jia, Du (101)
Garlic (*Allium sativum*)*	Liquid	0.005%, 0.01% and 0.02%	30 days	Allicin (98% purity, commercial product)	Large yellow croaker (*Larimichthys crocea*)	↑ Final weight ↑ Final length ↑ SGR ↑ survival rate	↑ Intestine total antioxidant capacity ↑ Intestine antioxidant enzymes (CAT, NO and NOS) activity ↓ Intestine transcriptional levels of pro-inflammatory genes	Improve the survival and growth of large yellow croaker larvae probably by promoting intestinal development, alleviating inflammation and enhancing appetite	Huang, Yao (102)
**Phytogenics combinations**									
Phytogenics combination	Essential oils	200 ppm	70 days + 15 days *N. girellae* challenge	Garlic and Lamiaceae-plants oils (N/I; commercial additive)	Greater amberjack (*Seriola dumerili*)	No effect	↑ Skin mucus lysozyme activity 15 days post *N. girellae* challenge ↑ Piscidin gene expression in skin pre-challenge ↑ Proinflammatory cytokines (*tnf-α* and *il1-β*), AMPs (*hep* and *cath*), immunoglobulin (*IgT*), complement protein (*c3*) T-cells marker (*cd8*) and mucin (*muc-2*) gene expression in skin post-challenge ↓ *Casp3* gene expression in skin post-challenge	Facilitates the immunological response of skin once the parasite is fixed, generating a hostile microenvironment in skin and lowering the parasite load	Fernández-Montero, Torrecillas (16)
Phytogenics combination	Essential oils	0.3 g kg^-1^	8 weeks + 2 weeks hypoxia challenge (Total 10 weeks)	Cinnamaldehyde, thymol and carvacrol	Nile tilapia (*Oreochromis niloticus*)	↓ Hepatosomatic index	↑ Intestine villi density ↓ Intestine malondialdehyde content	Positive effects of digestion and antioxidative capacity	Ning, Zhang (103)
Phytogenics combination	Microencapsulated essential oils	0.5%	65 days	Garlic essential oil (N/I), carvacrol and thymol (Commercial additive)	Gilthead seabream (*Sparus aurata*)	No effect	↑ Skin mucus inhibitory activity against *V. anguillarum* and *P. anguilliseptica* ↓ Cortisol in skin mucus ↑ Regulation of genes associated to the secretory pathway in skin ↑ Regulation of genes associated to non-specific immune response in skin ↑ Regulation of genes coding for oxidative stress enzymes in skin	Beneficially affects the skin and mucus immune and stress parameters, suggesting the stimulation and recruitment of phagocytic cells and a reduction in the fish allostatic load	Firmino, Fernández-Alacid (14)
Phytogenics combination	Microencapsulated essential oils	0.5%	65 days	Garlic essential oil (N/I), carvacrol and thymol (Commercial additive)	Gilthead seabream (*Sparus aurata*)	No effect	↑ Regulation of genes related to processes of proteolysis and inflammatory modulation, immunity, transport and secretion, response to cyclic compounds, symbiosis, and RNA metabolism in the mid-anterior intestine No effect upon alpha diversity of bacterial communities in the anterior and posterior intestinal tract sections ↓ Spirochaetes phylum in the posterior intestine ↑ *Photobacterium* and *Corynebacterium* genera in the anterior intestine ↓ *Comamonas* in the anterior intestine, and *Paracoccus*, *Prevotella* and *Rothia* genera in the posterior intestine ↓ Bacterial sequences related to carbohydrate and drug metabolisms, and membrane transport ↑ Bacterial sequences related to glutathione and lipid metabolisms, naphthalene degradation and sulphur relay system Evidence of host-microbial co-metabolism	The activation of leukocytes and crosstalk between gut and microbiota are suggested to regulate the inflammatory response induced by the additive	Firmino, Vallejos-Vidal (12)
Phytogenics combination	Microencapsulated essential oils	0.5%	65 days + 39 days *S. chrysophrii* challenge (total 104 days)	Garlic essential oil (N/I), carvacrol and thymol (Commercial additive)	Gilthead seabream (*Sparus aurata*)	↓ *S.* *chrysophrii* total parasitation	↑ Regulation of genes related pro-inflammatory immune response arbitrated by degranulating acidophilic granulocytes, sustained by antioxidant and anti-inflammatory responses in gills ↑ Carboxylate glycoproteins containing sialic acid in mucous and epithelial gill’s cells	Promotes gill mucosal immunity and reduces gill ectoparasite incidence	Firmino, Vallejos-Vidal (15)
Phytogenics combination	Essential oils	0.02%	9 weeks + 1 week stress and *V. anguillarum* challenge	Garlic and Lamiaceae-plants oils (N/I; commercial additive)	European sea bass (*Dicentrarchus labrax*)	↑ Survival against *Vibrio anguillarum* when stress-challenged	↑ Skin mucus lysozyme activity when stress and bacterial challenge	Attenuate the fish physiological response to stress increasing resistance to *Vibrio anguillarum* infection	Serradell, Torrecillas (104)
Phytogenics combination	Essential oils	0.02%	63 days	Garlic and Lamiaceae-plants oils (N/I; commercial additive)	European sea bass (*Dicentrarchus labrax*)	No effect	↓ Shannon alpha diversity of mucosa-associated microbiota ↑Clostridiales order in intestinal content ↓ Coliforms and Vibrionales allochthonous microbiota	Reduction of orders containing potentially pathogenic species for fish, and enrichment of gut microbiota composition with butyrate producer taxa	Rimoldi, Torrecillas (105)
Phytogenics combination	Essential oils	200 ppm	63 days + 7 days stress and *V. anguillarum* challenge	Garlic and Lamiaceae-plants oils (N/I; commercial additive)	European sea bass (*Dicentrarchus labrax*)	No effect	↓ Posterior intestine fold area covered by goblet cells ↓ Posterior intestine goblet cells area ↑ Intestine mucus coverage post-challenge	Protective effect focused mainly on the preileorectal valve region	Torrecillas, Terova (13)
Phytogenics combination	N/I extract	6.0 g kg^-1^	30 days + 10 days crowding stress (40 days total)	Saint John’s wort (*Hypericum perforatum*, Hypericacea), lemon balm (*Melissa officinalis*, Lamiaceae) and rosemary (*Rosmarinus officinalis*, Lamiaceae) mixed at a ratio 3:2:1	Atlantic salmon (*Salmo salar*)	No effect	↓ Gut lipid peroxidation	Improves the gut antioxidant status	Reyes-Cerpa, Vallejos-Vidal (106)
Phytogenics combination	Powder	1%	45 days	Savory (*Satureja khuzestanica*, Lamiaceae) 0.5% and Onion (*Allium cepa*, Alliaceae) 0.5%	Common carp (*Cyprinus carpio*)	N/I	↑ Intestinal lactic acid bacteria	Improves intestinal health	Mousavi, Mohammadiazarm (87)
Phytogenics combination	Essential oils	0.06, 0.2, 0.4 and 0.8 g kg^-1^	6 weeks	Thymol and carvacrol (1:1; commercial additive)	Hybrid tilapia (*O. niloticus* ♀ × *O. aureus* ♂)	N/I	↑ Posterior intestine villus height ↑ Posterior intestine goblet cell count per villus ↑ Anterior intestine intraepithelial leucocytes ↓ Distal intestine intraepithelial leucocytes ↑ OTUs, and PD whole tree and Chao1 diversity indexes ↓*Thermi* phylum and *Bacteroides*, *Candidatus Cardinium*, and *Leptospirillum* genera	Affect the immunity primarily through a direct effect on host tissue but also has an indirect effect mediated by microbial changes	Ran, Hu (107)
Phytogenics combination	Essential oils	100 ppm	9 weeks	25% thymol and 25% carvacrol (commercial additive)	Gilthead seabream (*Sparus aurata*)	↓ FGR	↑ Intestine mucosal foldings ↑ Intestine enterocytes ↑ Intestine goblet cells ↓ Expression of genes related to cell differentiation and proliferation, intestinal architecture and permeability, immunosurveillance, such as cytokines, in the intestine	Induce an anti-inflammatory and anti-proliferative transcriptomic profile with probable improvement in the absorptive capacity of the intestine	Perez-Sanchez, Benedito-Palos (108)

SGR, Specific Growth Rate.

FCR, Feed Conversion Ratio.

FGR, Feed Gain Ratio.

OTU, Operational Taxonomic Unit.

*Indicate the putative plant species with high content of the referred bioactive compound. N/I, not identified or not assessed. Studies reporting the application of plant extracts or related compounds as bath treatments or evaluating bactericidal or antiparasitic effects *in vitro* were excluded from the selection. The table omits systemic immunity-related results, digestive enzymes or other complementary analysis performed within each study. Blends with other components besides the selected group of plants, terpenes or organosulfur compounds were excluded as well.

### Effect of Dietary Terpene Phenolic Compounds Upon Fish Mucosal Immunity

Phenolics and terpenes are a group of volatile plant-derived bioactive compounds with medicinal and biotechnological value that constitute the dominant fraction of the essential oils derived from aromatic plants (3). The monoterpenes carvacrol and its isomer thymol are the most studied phenolic compounds, representing the major components of the essential oils from several aromatic plants of the Lamiaceae family like the oregano (*Origanum vulgare*) and thyme (*Thymus vulgaris*) (109, 110). These compounds are particularly studied and recognized for their bactericidal activity, since their lipophilic character act as bacterial membrane permeabilizers with cytotoxic effects upon bacterial structure and function, leading to membrane expansion, fluidity and permeability, disturbance of the membrane-embedded proteins, respiration inhibition and alteration of ion transport. In addition, carvacrol and thymol were demonstrated to act as quorum sensing (QS) inhibitors, reducing bacterial biofilm formation. Carvacrol in particular, is able to inhibit bacteria motility, collapsing the proton-motive force, depleting the ATP pools and preventing the synthesis of flagellin (111). This bactericidal property highlights the ability of these compounds to potentially modulate mucosal tissues associated microbiota.

Together with their well-studied bactericidal potential, these phenolic compounds are described to potentially improve the integrity of the mucosal tissues due to their observed antioxidant, anti-inflammatory and consequent immunomodulatory properties in the gastrointestinal mucosa of several animal models (112). The reported strong antioxidant activity of carvacrol and thymol rely on their ability to scavenge free radicals, inhibiting reactive oxygen species (ROS) and other oxygen radicals generated in cells and tissues (113). By contrast, high concentrations may display antagonistic pro-oxidant effects (113). This dose-dependent antagonistic activity evidences the importance of correctly define their administration doses in order to obtain the desired results with regard to their immunomodulatory properties.

Regarding their anti-inflammatory potential, carvacrol and thymol appear to interfere with the NF-*k*B and MAPK pathways, modulating the expression of pro-inflammatory and anti-inflammatory cytokines (114, 115). It is commonly speculated that the anti-inflammatory properties of plant-derived bioactive compounds, such as carvacrol and thymol, may be attributed to their capacity to inhibit TLR-mediated NF-*k*B signaling pathways (116, 117). Furthermore, evidence that both carvacrol and thymol play a role in the chemosensory system through the activation of transient receptor potential (TRP) cation channels exist (118, 119). In higher vertebrates, TRP channels are widely expressed in several cellular types that includes most of the mucosal components. Through the maintenance of the intracellular calcium homeostasis, these channels are known to regulate several cell functions, such as stimuli perception, inflammatory molecules production and secretion, migration and even phagocytosis (120–122). Carvacrol and thymol are known to activate both the receptor TRPA1 (119) and the receptor TRPV3 in mucosal tissues, elevating cytosolic Ca^2+^ concentration in epithelial cells (118, 123). In fish, together with TLRs, the activation of TRP channels has been demonstrated to modulate the inflammatory processes through the activation of the TRP/Ca^2+^/TAK1/NF-*k*B signaling pathway (124). This suggests that a TRP channel mediated cellular activation may underlie the immunomodulatory properties of these bioactive compounds.

The health promoting effects of oregano, thyme and their derivates in fish have been recently reviewed (55, 125). Concerning their impact upon fish mucosal immunity, several nutritional studies have described beneficial effects of phytogenics derived from oregano, thyme and other plants of the Lamiaceae family upon the mucosal tissues in several fish species (**Table 1**). Most of them have reported an increase in skin mucus immune markers and/or skin mucus bactericidal activity (56–58, 65, 71, 73–79). The repeatedly evaluated markers were lysozyme, alkaline phosphatase, complement and protease activities, total immunoglobulin and protein content in fish skin mucus, as well as its *in vitro* bactericidal potential against bacterial pathogens. Several of these studies also described an improvement in key performance indicators, such growth, feed efficiency and survival against pathogenic bacterial challenges (56, 58, 65, 71, 73–79). Besides the assessment of key performance indicators and general immune markers in skin mucus, few studies have tried to explain and characterize the immunomodulatory mechanisms underlying such responses neither which specific compounds might be exerting such effects.

Carvacrol, thymol, p-cymene and γ‐terpinene are identified as the predominant bioactive compounds of most of the members of the Lamiaceae family considered in this review, which were mainly found in oregano and thyme. In addition, peppermint, rosemary and basil contain preponderant concentration of other bioactive compounds such menthol, eugenol and 1,8-cineole (**Table 1**). Interestingly, some studies have reported carvacrol and 1,8-cineole presence in the fish skin mucus (63, 81). This phenomenon of bioactive compounds efflux through skin could be responsible for the immunomodulatory and antimicrobial effects observed in the fish skin mucus. However, most of the reviewed studies did not report the phytogenics composition neither the assessment of their translocation through mucus.

Some studies have also reported a protective effect through the reduction in gills’ histopathological lesions induced by toxic element exposure or pathogenic challenges (62, 66, 85). Contrarily to the studies describing the effects of phytogenics upon skin mucus secretion and their immunomodulatory potential, their impact upon the GIALT is very scarce, being mostly limited to histological observations. Similarly, studies on the impact of phytogenics upon the intestine are commonly focused on evaluating alterations in morphoanatomical parameters such as an increase in villus length, width and goblet cells count, which are usually associated to improvements in fish growth performance (59, 60, 64, 84, 85). Some studies have also reported the modulation in the number of intestinal lymphocytes (70, 72). Other authors have described a positive impact upon the gastrointestinal activity of humoral immune markers, such lysozyme (86), the activity of antioxidant enzymes and oxidative markers (67, 106) or the down-regulation of the expression of pro-inflammatory genes, such *tnfα* and *tnfβ* (61). Besides, the beneficial impact of phytogenics administration upon the intestine microbiota composition was also suggested (61, 87). Nonetheless, analysis described were constantly incomplete in terms of mucosal immune response evaluation, since limited classical immune or oxidative markers were assessed in each of the above-mentioned studies. Similarly, microbiota studies were often restricted to a particular group of bacteria, such lactic bacteria, failing to properly characterize microbiota functionality and modulation by the experimental diets; thus, resulting in partial and biased conclusions when assessing the regulatory effects of functional feed additives on mucosal tissues.

In addition, some studies reported no effect of the administrated phytogenics upon mucosal parameters (68, 69, 80, 82, 83). Such discrepancies among studies evaluating a particular plant extracts may be due to the diversity of the referred studies in terms of experimental design, fish species selected, plant origin, supplemented form and inclusion level of phytogenetics, among others. This miscellaneous of studies and the lack of protocols allowing appropriate additive and animal testing, highlights the urgent need to standardize the experimental designs and procedures in order to properly evaluate these compounds under *in vivo* conditions and acquire relevant data for their further development and general and safe use.

The effect of some single specific bioactive compounds related to aromatic plants of the Lamiaceae family, such thymol or carvacrol, upon mucosal tissues were also reported (**Table 1**). Although these studies have the advantage to associate a specific mucosal response to the administration of a specific compound, once again it is observed that most of them only reported the analysis of few immune and oxidative markers (97, 98, 101, 103), or a limited microbiological examination (99, 100). In fact, few studies were observed to apply complementary analysis, or achieved to successfully discuss the multifactorial impact exerted by such nutritional strategies upon mucosal tissues (107, 108).

Although the overall data suggest the therapeutic potential of phytogenics derived from Lamiaceae family of plants in aquafeeds, especially of their associated terpene phenolic compounds, unfortunately none of the studies has proposed accurate mechanisms that could be responsible for the broad effects of these metabolites described upon fish mucosal tissues. Despite the lack of reliable information for aquaculture relevant fish species, it is possible that the above-mentioned antimicrobial properties of these compounds, their free radicals’ scavenging ability, along with their aptitude to activate TRP channels that modulate inflammatory processes may underlie the immunomodulatory properties and microbiota modulation described in different mucosal tissues.

### Effect of Dietary Organosulfur Compounds Upon Fish Mucosal Immunity

The main constituents of extracts and essential oils from *Allium* sp., such as garlic (*Allium sativum*, Alliaceae) and onion (*Allium cepa*, Alliaceae), are sulfur‐containing compounds. This group of bioactive substances comprises alliin, allicin and its derived bioactive compounds like ajoene, diallyl trisulfide (DATS), diallyl disulfide (DADS), diallyl sulfide (DAS) and allyl methyl disulfide, commonly termed as organosulfur compounds. These organosulfur compounds are the responsible for the recognized antioxidative, antimicrobial, antifungal and antiparasitic properties of garlic (126). Allicin (S-allyl-2-propenyl thiosulfinate) is usually the main biologically active component of garlic and related species; however, it is highly unstable under physiological conditions; thus, quickly being transformed into its organosulfur derivates, which also exhibit therapeutic properties (127–129).

Organosulfur compounds have been particularly studied for their antiparasitic activity. Among them, ajoene was described to interfere with parasite and host cell membrane protein and lipid trafficking, with irreversible detrimental consequences for the parasite (130). This is of special relevance since teleost mucosal tissues are known to have a high constitutive expression of Th2 markers that indicate a skewed immune response targeted against parasites (131). Regarding their bactericidal properties, the organosulfur compounds can penetrate the bacterial cell membranes, cause changes in the structure of thiol (-SH) containing enzymes and proteins, and lower the expression of important genes involved in the QS in bacteria, inhibiting the growth of both Gram-positive and Gram-negative bacteria (132). The higher the number of sulfur atoms present in the compounds, the more is its bactericidal activity (133). Therefore, the administration of organosulfur-containing phytogenics may induce important changes in the fish mucosal-associated microbiota with potential effects upon the mucosal immunity.

The detoxification and chemoprotective benefits from various organosulfur compounds have been associated to their ability to scavenge free radicals and selectively enhance or suppress the levels genes or proteins of several antioxidant enzymes, such as cytochrome P450 enzymes or glutathione S-transferase (GST) (134), exerting a direct effect upon immune cells (135). In this line, their anti-inflammatory activity upon immune and intestinal epithelial cells was associated to the inhibition of ROS production and the modulation of the NF-*k*B and MAPK signaling pathways (136, 137). Some organosulfur compounds, such allyl sulphides, were also observed to increase the levels of anti‐inflammatory H_2_S in intestinal epithelial cells, promoting mucosal integrity, tissue repair and stimuli perception (138). In accordance, organosulfur compounds, such as DADS, are also donors of H_2_S, whose positive effects upon the intestinal health could be also produced through the modulation of the enteric microbiota (139). In addition, organosulfur compounds were observed to promote mucin expression in human airway epithelial cells, being suggested to improve the mucosal epithelial barrier function (140, 141).

Conversely, these organosulfur compounds have been also reported to stimulate inflammatory immune responses, promoting the release of pro-inflammatory cytokines, enhancing the proliferation of lymphocytes, macrophage phagocytosis and modulating the infiltration of immune cells (142). For instance, it was demonstrated that some allyl-containing organosulfur compounds directly activate Ca^2+^ flux in neutrophils augmenting their phagocytic function and consequent ROS production. In parallel, other compounds are able to inhibit spontaneous ROS production by neutrophils (143). This apparent antagonistic effect evidences the pleiotropic protective effects of garlic extracts and essential oils, being simultaneously capable of inducing immune responses and anti-inflammatory counteractions. Moreover, as previously suggested for the mode of action of terpene phenolic compounds, organosulfur compounds are also able to activate TRP channels, TRPA1 and TRPV1 channels in particular (144–146), suggesting the Ca^2+^ induced cellular immune activation (143).

Garlic has been for long studied and recognized for its benefits as growth- and flesh quality-promoting effects in cultured fish, as well as for its antibacterial and antiparasitic properties (51). However, there is scarce information regarding the activity of garlic-derived organosulfur bioactive compounds upon fish mucosal tissues. The synthesis of the results from several studies reporting the health promoting properties of phytogenics derived from *Allium* sp. are shown in **Table 1**. Similar to studies testing phytogenics from Lamiaceae family origin, the evaluation of the supplementation of phytogenics derived from *Allium* sp., also focuses on few immune markers in skin mucus (88, 93), histopathological observations or some inflammatory and oxidative markers in gills (89, 96) and intestine (90, 94–96, 102), or incomplete microbiological examination that lacks in-depth the functional interpretation of their mode of action at cellular level (87, 90, 92).

Overall, studies reporting the effect of the administration of phytogenics derived from *Allium* sp. upon fish mucosal tissue suggest the health-promoting potential of the organosulfur compounds that characterize this group of plants. However, there is currently no robust studies under a pharmacological point of view that intent to demystify the accurate mechanisms responsible for the effects described on fish mucosal tissues, whose lack of reliable information critically restrains their application as potential functional feed additives in aquafeeds. Similar to the terpene phenolic compounds, the organosulfur compounds have also recognized antipathogenic and antioxidant properties, in addition to their common ability to activate TRP channels that modulate inflammatory processes. Since in higher vertebrates dermal emission of organosulfur compounds were demonstrated after garlic ingestion (147). In this sense, the efflux of organosulfur compounds through the integument could be also playing a critical role in the recurrently reported effects of dietary garlic and other aromatic plants in fish mucus, as previously referred for carvacrol and 1,8-cineole.

### Effect of Combinations of Phytogenics Derived From Lamiaceae and *Allium* sp. Upon Fish Mucosal Immunity

Some studies have reported the beneficial effects of the combination of Lamiaceae and *Allium* sp. phytogenics upon fish mucosal tissues (**Table 1**). For instance, in European seabass (*Dicentrarchus labrax*) a combination of garlic and essential oils from plants of the Lamiaceae family promoted skin mucus lysozyme activity and fish survival against *V. anguillarum* when exposed to a confinement stress (104). In greater amberjack (*Seriola dumerili*), the same additive induced an up-regulation of a set of immune related genes in the skin in response to a monogenean parasite *Neobenedenia girellae* infection (16). In accordance, a blend of garlic essential oils, carvacrol and thymol was also reported to positively impact both gilthead seabream (*Sparus aurata*) skin mucus in terms of bacterial inhibition capacity against fish pathogens and decrease of stress markers, whereas the transcriptional analysis suggested the stimulation of the secretory pathway possibly associated to humoral immune molecules secretion into mucus and activation of phagocytic cells (14). The same blend was reported to regulate the transcription of genes related to immune response in gills, which was mediated by granulocytes, as well as sustaining both anti-inflammatory and antioxidative responses. In addition, the above-mentioned study revealed that the tested phytogenic compounds promoted the presence of sialic-acids containing glycoproteins in both epithelial and mucous cells, which globally resulted in a decrease in the intensity of gills’ infestation by the monogenean ectoparasite *Sparicotyle chrysophrii* in gilthead seabream (15). Both, referred phytogenics combinations were observed to positively affect the gut health status of those fish species by improving the protective intestine mucus coverage post-challenge (13), regulating the intestine immune transcription (12) and modulating their intestinal microbiota (12, 105).

**Figure 1:**
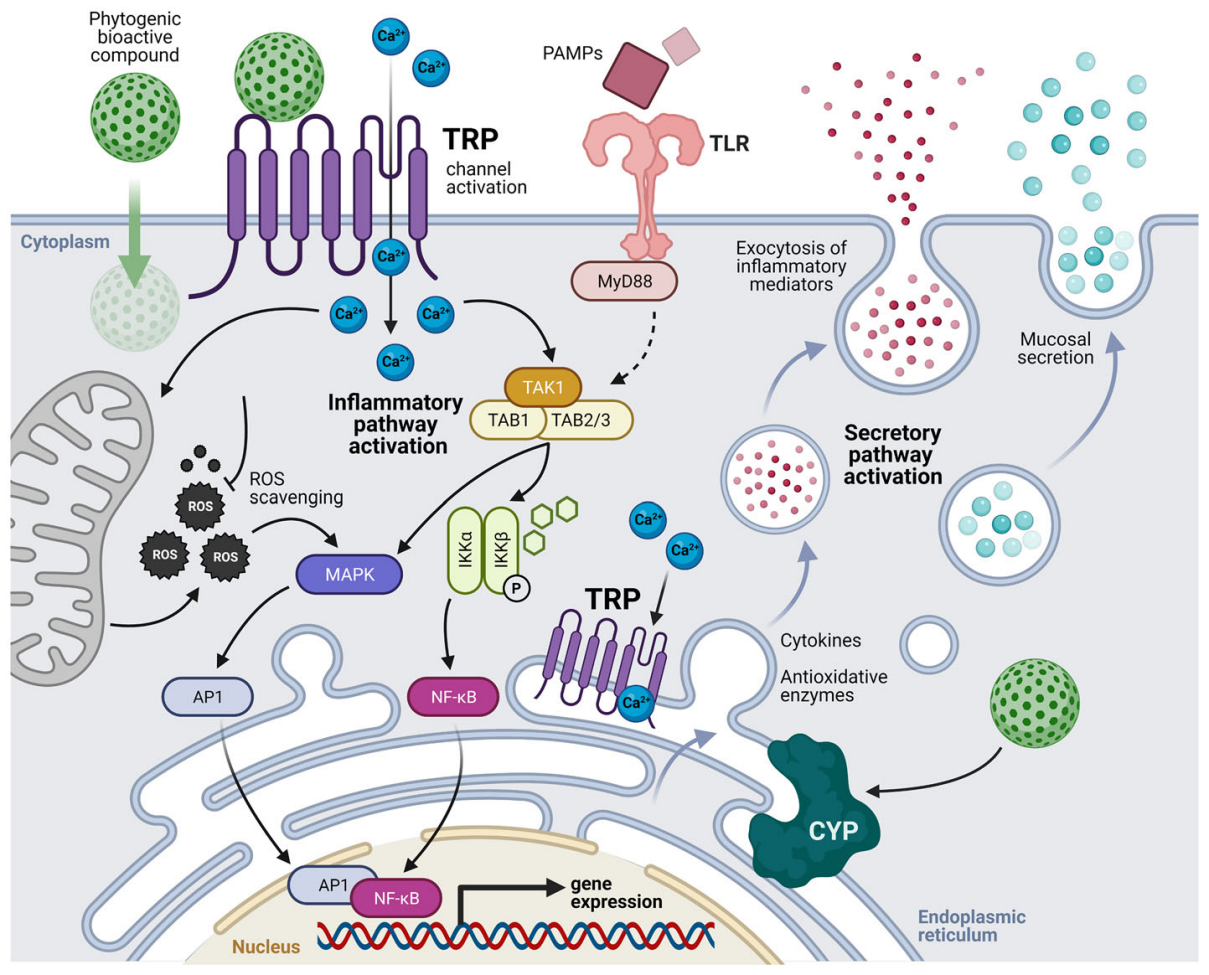
Suggested mechanisms of cell activation by the transient receptor potential (TRP) cation channels mediated by phytogenics’ bioactive compounds in mucosal-associated lymphoid tissues (MALTs). Bioactive compounds activate TRP channels leading to intracellular Ca^2+^ increase and non-canonical activation of the TAK complex. In parallel, stimulation by pathogen-associated molecular patterns (PAMPs) may facilitate the activation of TLR and TRP signaling pathways. Modified from Galindo-Villegas, et al. (124). TLR, toll-like receptors; MyD88, myeloid differentiation primary response 88; TAK, transforming growth factor beta (TGFβ) activated kinase; TAB, TGFβ activated kinase binding protein; ROS, reactive oxygen species; NF-*k*B, nuclear factor kappa-B; IKK, inhibitor of NF-*k*B kinase; MAPK, mitogen-activated protein kinase; AP1, activator protein 1; CYP, cytochromes P450; P, phosphorylation.

According to the above-described studies and the acknowledged properties of these phytogenics’ bioactive compounds, we suggest that the mechanisms of cell activation that may be responsible for the mucosal immune-related responses are mediated by the activation of TRP cation channels in both immune and epithelial cells of mucosal tissues. The bioactive compounds may activate TRP channels leading to intracellular Ca^2+^ increase and the activation of the TAK1/MAPK/NF-*k*B signaling pathways, modulating the expression of pro-and anti-inflammatory cytokines, and antioxidative enzymes such as cytochrome P450. In parallel, stimulation by pathogen-associated molecular patterns (PAMPs), who might be also modulated by the antimicrobial properties of these compounds, may facilitate the activation of TLR and TRP signaling pathways; thus, amplifying the mucosal immune responses. Moreover, the bioactive compounds are also suggested to passively diffuse across the cell membrane, scavenging ROS that contribute to the inflammatory pathways, and interacting with TRP channels of the endoplasmic reticulum, potentially stimulating the secretory pathway. The above-described mode of action of phytogenics derived from Lamiaceae family and *Allium* sp. at the level of the main mucosal lymphoid tissues in fish is depicted in **Figure 1**.

**Figure 2:**
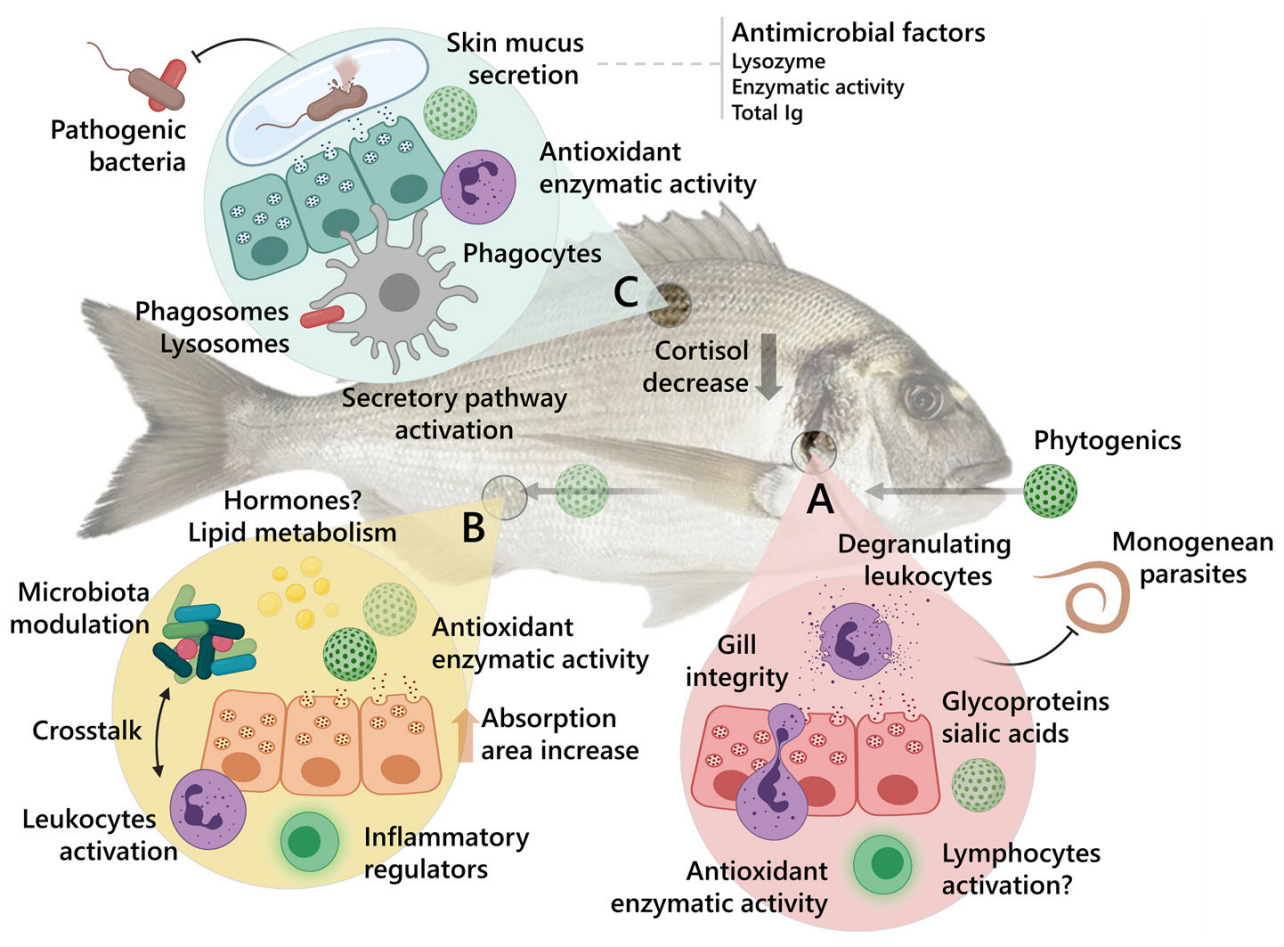
Summary of the proposed mode of response induced by phytogenic bioactive compounds in fish mucosal tissues. The effects against representative types of common pathogens on the most studied mucosal-associated lymphoid tissues in fish so far are represented in the figure. **(A)** Gills. **(B)** Gut. **(C)** Skin. Gilthead seabream (*S. aurata*) was used in the figure as a representative aquaculture-relevant fish.

In addition, a summarized representation of the potential mucosal immune responses induced by the dietary administration of terpene and/or organosulfur based phytogenics and their effects against fish pathogenic organisms is suggested in **Figure 2**. In this representation, the holistic perspective of the compounds’ effects upon the most studied mucosal-associated lymphoid tissues in fish so far – gill, gut, and skin – as targets for oral immunostimulation is highlighted through the stimulation of both humoral and cellular immunity, mucosal secretion, microbiota modulation and other potential physiological and metabolic responses.

## Future Perspectives

Feeding the projected world population by 2050 in a sustainable way is a great challenge, in which aquaculture is predicted to supply the majority of aquatic dietary protein. For that, the implementation of novel policies and production system approaches targeting effective health management and animal welfare are mandatory (1). Moreover, indiscriminate prophylactic use of antibiotics associated to intensive aquaculture practices can still be observed among some of the major aquaculture producing countries, as it has been recently reviewed (148, 149). However, in 2022, several countries, including the EU will prohibit all forms of routine antibiotic use in farming, including preventative group treatments which highlights the necessity for the development of more sustainable alternative treatments (150). Under this context, the market for sustainable products and feed additives is increasingly growing and the number of studies on the use of a wide variety phytogenics as sustainable tools to be implemented in aquaculture production has increased dramatically in the last decade (5). The global market of phytogenic feed additives including major tier I and II suppliers was estimated on 753M USD in 2020 and it is projected to reach 1,098M USD by 2025 (151).

Although limitations in testing and reproducing studies using dietary immunostimulants have been pointed out since long ago (152–155), the current knowledge on the pathways and mechanisms followed by these compounds at the cellular level is still neglected. A large selection of experimental designs, fish species, phytogenics tested and diet composition fails in the association of selected bioactive compounds to specific effects. Moreover, it is important to consider the difficulties to carry out comparison among the available studies because of the different assays, testing methods, different extraction procedures of plant essential oils or extracts, and the intrinsic variation in chemical phytoconstituents in plants due to different agroclimatic conditions, harvesting season and plant phenotype. This essential oils or extracts consist of a variable mixture of different bioactive molecules that area generally not characterized, and are administrated through variable periods of time, dietary doses and forms in different fish species that are generally randomly selected. In addition, a considerable number of these studies provide little or partial information regarding the effect of a given immunostimulant, since their definitive efficiency assessment rely on the evaluation of basic biochemical parameters that are to some extent obsolete if compared against the actual state-of-the-art. Based on the former idea, diverse omics tools available play a fundamental role for proper understanding and characterization of their mode of action in mucosal tissues at cellular level. Together, these factors question the reproducibility and safety of a large number of studies available and limits the use of several of those proposed substances in commercial functional feeds.

In this regard, we propose that the study of purified bioactive compounds may represent a viable solution to circumvent variability, and the biological mode of action of isolated compounds should be primarily assessed *in vitro* under variated settings, before moving forward to *in vivo* trials. However, it is important to consider that the biological activities of essential oils or extracts cannot be attributed to a single compound or to a unique specific mechanism, since their multi-component properties exert greater biological activity when compared to the major components alone, whose function is probably regulated by the synergy with limiting compounds (8). In this light, essential oils and extracts from different plants have been explored for their potential as resistance modifying agents (156). While their chemical complexity may represent a clear advantage in terms of reducing the risk of inducing bacterial adaptation and resistance to single compounds, or even promoting a wider antibacterial activity, the use of blends of phytogenics makes difficult to proper characterize their mode of action. Therefore, when developing such additives, the formulation of combined purified compounds through the correct and soundly *in vitro* functional characterization to obtain potential synergies is recommended. Moreover, long-term studies assessing whether the bioactive compounds, single or combined, induce bacterial tolerance, transmissible adapted resistance or any other change on a large scale should be implemented and the effects on both beneficial and pathogenic bacteria determined through *in vitro*, *in silico* and finally *in vivo* approaches (103, 157).

While several phytogenics have been proved remarkably efficient in promoting mucosal fitness (9), little efforts have been made to elucidate the underlying pharmacokinetics and immunostimulatory mechanisms of tested compounds upon the MALTs, with few *in vitro* studies published to date. It should also be highlighted that occasionally *in vitro* studies do not accurately translate into predictable responses *in vivo* (83); thus, both *in vitro* and *in vivo* studies should be performed whenever possible. This lack of complementary information supports the demand for additional profound research on the fate and length in which particular phytogenic compounds act, which is crucial for further developing functional additives and their application in an industrial context. Although the specific mechanisms behind the observed fish mucosal physiological responses are still poorly described, it is possible that cellular pathways involving the activation of TRP receptors by the bioactive compounds might be responsible for the reported mucosal immune responses. Besides, this response might be potentiated by the PAMP-induced activation of the TLR cell-signaling cascade, as synthetized in **Figure 1**, which would explain the fish improved ability to cope with pathogenic challenges. Thus, it is advisable in nutritional dose-response evaluating phytogenics to evaluate changes in expression in TRP receptors as well as gene markers of the TAK1/MAPK/NF-*k*B signaling pathways in order to provide insight into their mode of action at mucosal level.

Another limitation that should be taken into account when testing phytogenics is that most plant-derived bioactive compounds are either volatile and/or susceptible to rapid degradation in the stomach where acid digestion takes place, with consequent low availability at the intestinal level or uncontrolled changes in the dose of administration. Hence, to overcome this limitation and minimize potential losses, controlled releasing techniques, such as encapsulation or other coating technologies, can be used to improve the proper delivery of phytogenics. This technology allows a prolonged absorption and local availability of the bioactive compounds along the gastrointestinal tract, ultimately increasing their beneficial impact upon the host (158, 159). Moreover, encapsulation protects phytogenics from environmental degradation, such as from light, temperature and/or pH variations, and eventually playing an important role in their palatability, masking the potential pungency associated to some compounds that otherwise can affect feed intake (158). It is important to highlight that most of the studies considered in this review did not take into consideration those aspects, administrating phytogenics as powder forms, hydroethanolic extracts or dissolved solutions without proper assessment of their potential biodegradation during feed storage or along the gastrointestinal tract. The overall limitations identified in most of the currently available studies assessing fish immunity leads to the stigmatization of phytogenics application, in which compounds with high pharmacological value are labeled under the “medicinal plants” or “herbalism” pseudoscience stigma, with disbelieving scientific evidence. This represents a major restriction for the development of effective phytogenics at commercial scale.

## Conclusions

Overall, it is fundamental that the efforts made in the research for sustainable prophylactic tools to boost host’s immune condition, stress resistance and pathogenesis prevention will culminate on reliable administration strategies for the aquaculture sector. Among the most studied group of natural bioactive compounds, both terpenes and organosulfur compounds have been suggested to display antimicrobial, antioxidant, anti-inflammatory and immunomodulating activities, with the potential of improving fish mucosal barrier function and integrity. Although they comprise a promising group of phytogenics for aquafeeds, an urgent update in the academical approach and experimental methodologies are needed to elucidate their pharmacokinetics and mode of action in depth. Therefore, in the present review we propose important molecular signaling pathways and hypothesize their involvement on the dietary immunomodulation in fish by the selected phytogenics.

## Author Contributions

JF and JG-V data analysis and visualization. JF wrote the draft. All authors contributed to the article and approved the submitted version.

## Conflict of Interest

JF is employed by TECNOVIT-FARMFAES S.L. FER-L is a senior research associate of the Consorcio Tecnológico de Sanidad Acuicola, Ictio Biotechnologies S.A. (Chile).

The remaining authors declare that the research was conducted in the absence of any commercial or financial relationships that could be construed as a potential conflict of interest.
